# Investigating the Relationship between Demographic, Radiological and Clinical Factors and In-Hospital Mortality of Non-Traumatic Subarachnoid Hemorrhage Before and After COVID-19 Pandemic: Mortality in Non-Traumatic SAH Before and After COVID-19

**DOI:** 10.31661/gmj.v14i.3866

**Published:** 2025-07-09

**Authors:** Seyed Hossein Aghamiri, Negar Mohamadi Khorasani, Hossein Farshadmoghadam

**Affiliations:** ^1^ Department of Neurology, School of Medicine, Shahid Beheshti University of Medical Sciences, Tehran, Iran; ^2^ School of Medicine, Shahid Beheshti University of Medical Sciences, Tehran, Iran; ^3^ Department of Pediatrics, Children Growth Research Centre, Research Institute for Prevention of Non-Communicable Disease, Qazvin University of Medical Science, Qazvin, Iran

**Keywords:** Subarachnoid Hemorrhage, Mortality, Covid-19, Risk Factors, Glasgow Coma Scale, Aneurysm, Angiography

## Abstract

**Background:**

Subarachnoid hemorrhage (SAH) is a life-threatening neurological condition that accounts for approximately 5% of all strokes. This study aimed to evaluate the demographic, clinical, and radiological factors associated with in-hospital mortality in patients with non-traumatic SAH and to assess potential differences before and after the COVID-19 pandemic.

**Materials and Methods:**

This retrospective analytical study was conducted on 177 patients with non-traumatic SAH admitted to Imam Hossein Hospital, Tehran, from November 2021 to December 2022. Diagnosis was confirmed by a neurologist using clinical presentation, imaging, and cerebrospinal fluid analysis. Patients were grouped based on discharge status (deceased vs. survived), and also classified into early- and late- pandemic subgroups based on their admission date. Comparative analyses and binary logistic regression were performed to identify predictors of in-hospital mortality.

**Results:**

Among 177 patients (mean age: 54.75 years), 36 (20.3%) died during hospitalization. Blood pressure, blood sugar, and level of consciousness at admission were significantly associated with mortality (P 0.05), as were disease severity (Hunt and Hess grade) and angiography status (P 0.001). No significant associations were found for age, sex, hospitalization duration, creatinine, platelets, hypertension, diabetes, or aneurysm characteristics (P 0.05). Logistic regression identified lower Glasgow Coma Scale (GCS) scores (P 0.001) and higher systolic blood pressure (P = 0.004) as independent predictors of mortality. Mortality was higher in the late-pandemic group (23.9% vs. 16.0%), though not statistically significant (P = 0.18).

**Conclusion:**

Lower GCS scores and elevated systolic blood pressure at admission were independent predictors of in-hospital mortality in patients with non-traumatic SAH. The COVID-19 pandemic period was associated with reduced use of angiography and a trend toward increased mortality, underscoring the importance of maintaining access to timely care during public health crises.

## Introduction

Subarachnoid hemorrhage (SAH) contributes to about 5% of all stroke events [1][2]. This hemorrhage occurs within the subarachnoid space, located between the arachnoid membrane and pia mater, and often leads to cerebral edema and neurological complications [3][4][5]. While traumatic brain injuries are a common cause, roughly 85% of SAH cases are non-traumatic in origin [1][2], with ruptured cerebral aneurysms accounting for over 80% of spontaneous events [6][7].

Elevated hemodynamic stress plays a critical role in aneurysm formation. Persistent hypertension can induce inflammation in vessel walls, promoting structural weakening and potential rupture [8]. Inflammatory cytokines have also been implicated in this process [9][10]. Besides hereditary predisposition, lifestyle factors such as hypertension, smoking, and alcohol use significantly contribute to aneurysm development [11][12].

Timely identification and management are crucial for favorable outcomes in non-traumatic SAH [13][14]. Diagnosis is more straightforward in patients presenting with severe symptoms [13][15], but subtle cases may be missed, resulting in delayed intervention and worse prognosis. Misdiagnosis rates during initial assessments have been reported at approximately 12% [16][17].

Importantly, the COVID-19 pandemic has profoundly affected healthcare systems worldwide, with potential consequences for the diagnosis, treatment, and outcomes of acute cerebrovascular events, including SAH. Limited hospital resources, delayed presentations, and hyperinflammatory responses associated with SARS-CoV-2 infection may influence both the clinical course and prognosis of patients. Recent studies have highlighted altered patterns of aneurysm rupture and increased mortality during the pandemic period [18][19]. Therefore, assessing the impact of the COVID-19 era on SAH characteristics and mortality is essential for future preparedness and optimization of care. Given these challenges, the present retrospective study aimed to evaluate the relationship between demographic, radiological, and clinical factors and hospital mortality in patients with non-traumatic SAH, with a specific focus on differences before and after the COVID-19 pandemic.

## Materials and Methods

### Study Design

This retrospective analytical study was conducted on the medical records of 177 patients diagnosed with non-traumatic subarachnoid hemorrhage (SAH) who were admitted to Imam Hossein Hospital between November 2021 and December 2022. The study protocol was approved by the Ethics Committee of the School of Medicine (IR.SBMU.MSP.REC.1401.228). Informed consent was obtained from all patients or their legal guardians. To explore the potential effects of the COVID-19 pandemic, patients were divided into two subgroups based on their admission date relative to the surge of the Omicron variant in Iran. The cutoff was set at March 1, 2022, which marked the peak of the Omicron wave. Patients admitted before this date were categorized as the "early-pandemic" group, and those admitted after as the "late-pandemic" group.

### Inclusion and Exclusion Criteria

Patients were included if they had a confirmed diagnosis of non-traumatic SAH and had complete medical records. Exclusion criteria included a history of trauma, coagulation disorders, other vascular abnormalities, and incurable comorbid conditions.

### Data Collection

Patient data were extracted from medical records after approval by the ethics committee. The diagnosis of SAH was made by a neurologist based on clinical presentation, imaging findings (CT, MRI, angiography), and cerebrospinal fluid examination where necessary. Information was collected from surgical notes, radiological reports, and follow-up documentation. Aneurysms were diagnosed using brain angiography, CT angiography, MR angiography, or direct intraoperative visualization in emergency surgeries. The Hunt and Hess scale was used to classify disease severity at admission. Patients who presented with hydrocephalus underwent ventricular drainage prior to angiography. Aneurysm clipping surgery was performed promptly following diagnostic confirmation. In addition to clinical variables, each patient’s admission date was recorded and used for temporal subgroup analysis (early vs. late-pandemic). Comparative analyses were conducted to identify differences in mortality rates, clinical characteristics, and treatment patterns between these two groups.

### Variables and Measurement

Data were recorded using a standardized checklist. Collected variables included age, gender, length of hospital stay, systolic blood pressure at admission, blood sugar at admission, serum creatinine, platelet count, level of consciousness at admission and on the seventh day, type and location of aneurysm, and whether angiography was performed. Patients were categorized into two groups (deceased vs. survived) based on their status at discharge. To identify independent predictors of in-hospital mortality, a binary logistic regression model was planned based on selected clinical and radiological variables. The dependent variable was hospital mortality status (deceased vs. survived), and independent variables included systolic blood pressure, blood sugar, Glasgow Coma Scale (GCS) at admission, Hunt and Hess grade, and angiography status. The COVID-19 era grouping (early/late-pandemic) was also included as an independent variable in logistic regression analysis to assess its potential association with in-hospital mortality.

## Statistical Analysis

Descriptive statistics, including mean and standard deviation, were used to summarize continuous variables, while frequencies and percentages were used for categorical data. The independent t-test was used to compare continuous variables between groups, and the chi-square test was applied for categorical variables. A subgroup analysis comparing early/late-pandemic admission cohorts was performed to evaluate temporal trends in clinical outcomes and mortality. Additionally, a binary logistic regression analysis was performed to identify independent predictors of in-hospital mortality. Odds ratios (OR) and 95% confidence intervals (CI) were reported for each variable in the model. A P-value of less than 0.05 was considered statistically significant. All statistical analyses were conducted using SPSS software version 26.0 (IBM Corp., Armonk, NY, USA).

## Results

**Table:** Table1. Comparison of clinical and laboratory findings based on patient outcomes

**Parameter**	**Deceased (n = 36) **	**Survived (n = 141) **	**P-value**
Age (years)	61.22 ± 14.45	53.10 ± 11.85	0.20
Duration of hospitalization (days)	14.06 ± 12.51	12.08 ± 13.58	0.37
Blood pressure at admission (mmHg)	150.36 ± 43.46	139.42 ± 25.97	0.001
Blood sugar at admission (mg/dL)	174.97 ± 90.64	146.18 ± 55.57	0.009
Serum creatinine (mg/dL)	1.11 ± 0.72	1.00 ± 0.36	0.18
Platelet count (×10³/μL)	207.21 ± 66.01	231.42 ± 67.55	0.84
GCS at admission	10.44 ± 4.78	14.45 ± 1.87	<0.001
GCS on day seven	6.18 ± 4.69	14.12 ± 2.37	<0.001

**Table:** Table2. Comparison of categorical variables based on mortality

**Variable**	**Deceased (n = 36) **	**Survived (n = 141) **	**P-value**
Sex (Male/Female)	19 / 17	67 / 74	0.58
Hunt and Hess Grade			<0.001
Grade 1	1 (0.6%)	25 (14.1%)	
Grade 2	9 (5.1%)	82 (46.3%)	
Grade 3	6 (3.4%)	28 (15.8%)	
Grade 4	12 (6.8%)	2 (1.1%)	
Grade 5	8 (4.5%)	4 (2.3%)	

**Table:** Table3. Radiological parameters and treatment approach

**Parameter**	**Deceased (n = 36) **	**Survived (n = 141) **	**P-value**
Treatment Type			
- Surgery	6 (3.4%)	28 (15.8%)	
- Endovascular	8 (4.5%)	26 (14.7%)	
- Medical	22 (12.4%)	87 (49.2%)	
Aneurysm Presence			0.67
- Aneurysmal SAH	15 (8.5%)	62 (35.2%)	
- Non-Aneurysmal SAH	21 (11.9%)	79 (44.9%)	
Aneurysm Location			0.79
- Anterior Circulation	12 (16.2%)	55 (74.3%)	
- Posterior Circulation	0 (0.0%)	2 (2.7%)	
- Combined (Anterior & Posterior)	1 (1.4%)	4 (5.4%)	
Angiography Performed			<0.001
- Yes	21 (11.9%)	121 (68.4%)	
- No	15 (8.5%)	20 (11.3%)	

**Table:** Table4. Logistic Regression Analysis for Predictors of In-Hospital Mortality

**Variable**	**Odds Ratio (OR) **	**95% Confidence Interval (CI) **	**P-value**
Systolic BP at admission	1.02	1.01 - 1.04	0.004
Blood Sugar at admission	1.01	0.99 - 1.02	0.172
GCS at admission	0.72	0.61 - 0.84	<0.001
Hunt and Hess grade	1.31	0.98 - 1.75	0.061
Angiography performed (Yes vs No)	0.79	0.34 - 1.83	0.580
COVID-19 period (early vs late)	1.51	0.68-3.37	0.310

Model fit: **χ²** = 42.87, **df** = 5, P < 0.001

**Table:** Table5. Comparison of patients early and late the COVID-19 pandemic

**Variable**	**early-** **pandemic** **(n = 81)**	**late-** **pandemic** **(n = 96)**	**P-value**
**Age (mean ± SD) **	54.4 ± 13.1	55.0 ± 12.4	0.68
**Gender (Male/Female) **	42 / 39	44 / 52	0.47
**Systolic BP at admission **	138.7 ± 28.6	144.3 ± 31.2	0.23
**Blood sugar at admission **	149.2 ± 63.5	155.3 ± 67.1	0.51
**GCS at admission **	13.9 ± 2.9	13.4 ± 3.2	0.19
**Hunt and Hess Grade ≥ 3 **	15 (18.5%)	23 (24.0%)	0.41
**Angiography performed (Yes) **	63 (77.8%)	58 (60.4%)	0.03
**In-hospital mortality **	13 (16.0%)	23 (23.9%)	0.18

Out of the 177 patients included in this study, 36 patients (20.3%) died during hospitalization, while 141 patients (79.7%) survived. Among the total patients, 86 were male and 91 were female. The mean age was 54.75 years. The average blood sugar level at admission was 152.39 mg/dL, and the mean serum creatinine level was 1.02 mg/dL. The average duration of hospitalization was 12.47 days. There were no statistically significant differences in age, duration of hospitalization, serum creatinine, or platelet count between the deceased and survived groups (P > 0.05). However, systolic blood pressure, blood sugar level at admission, and level of consciousness at admission and on the seventh day were significantly associated with in-hospital mortality (P < 0.05) (Table-1). The Hunt and Hess scale was strongly associated with mortality (P < 0.001); patients with higher grades had a higher risk of death. No significant relationship was found between mortality and gender (P = 0.58), history of hypertension, diabetes, smoking, or alcohol consumption (P > 0.05), although hypertension showed a trend toward significance. Regarding radiological data, there was no significant association between mortality and treatment type, aneurysm presence, or aneurysm location. However, angiography status was significantly associated with patient outcomes (P < 0.001), with more survivors having undergone angiography compared to non-survivors (Table-2 and Table-3).

Subgroup Analysis Based on COVID-19 Pandemic Period

To evaluate the potential impact of the COVID-19 pandemic, patients were divided into two groups based on their admission date relative to the peak of the Omicron variant wave in Iran on March 1, 2022. The early-pandemic group included patients admitted before this date (n = 81), and the late-pandemic group included those admitted after (n = 96).

• Among early- Pandemic patients, the in-hospital mortality rate was 16.0% (13 deaths).

• Among late- Pandemic patients, the mortality rate increased to 23.9% (23 deaths), though the difference did not reach statistical significance (P = 0.18).

Additionally, angiography was more frequently performed before the pandemic (77.8%) compared to after (60.4%) (P = 0.03), suggesting potential delays or limitations in diagnostic services during the pandemic.

No significant differences were observed between the two groups in terms of age, gender, blood sugar, or Hunt and Hess grade (P > 0.05). However, patients admitted late- Pandemic showed a slightly higher average systolic blood pressure and lower GCS scores at admission, although these were not statistically significant. (Table-5)

Logistic Regression Analysis

To further evaluate the association between clinical variables and in-hospital mortality, a binary logistic regression analysis was conducted. The model was statistically significant (χ² = 42.87, df = 6, P < 0.001, Table-4).

The COVID-19 period (early vs. late) was included as an independent variable but was not a statistically significant predictor of mortality (OR = 1.51, 95% CI: 0.68-3.37, P = 0.31).

Significant independent predictors of mortality included:

• Lower GCS at admission (OR = 0.72, 95% CI: 0.61-0.84, P < 0.001)

• Higher systolic blood pressure (OR = 1.02, 95% CI: 1.01-1.04, P = 0.004)

## Discussion

This study aimed to investigate the demographic, clinical, and radiological factors associated with in-hospital mortality in patients with non-traumatic subarachnoid hemorrhage (SAH). Our findings indicated that systolic blood pressure, blood sugar levels, level of consciousness at admission and on day seven, Hunt and Hess grade, and angiography status were associated with patient outcomes.

Logistic regression analysis further revealed that lower GCS at admission and higher systolic blood pressure were independently associated with increased risk of mortality. These results highlight the prognostic importance of both neurological and hemodynamic parameters in SAH. Similar findings have been reported in prior studies, which underscore that impaired consciousness and blood pressure dysregulation are strong predictors of poor outcomes in SAH patients [18][19].

Although univariate analyses showed significant relationships between Hunt and Hess scores, blood sugar levels, and mortality, these variables did not retain significance in multivariate analysis. Hunt and Hess score exhibited a trend toward significance, suggesting its role may be influenced by co-variates. Previous studies have consistently supported this grading system as a reliable indicator of disease severity and prognosis [20][21].

In line with existing literature, angiography status was significantly associated with survival in our univariate analysis. However, this association was not statistically significant in the multivariate model, potentially due to confounding factors such as clinical stability or timing of intervention [22][23].

Importantly, this study also explored the potential impact of the COVID-19 pandemic period on SAH outcomes by comparing patients admitted during the early-pandemic (before March 1, 2022) and the late-pandemic (after March 1, 2022), which approximately corresponds to the period before and after the peak of the Omicron variant wave in the region.

Although in-hospital mortality was higher in patients admitted during the late-pandemic period (23.9%) compared to the early-pandemic period (16.0%), the difference was not statistically significant. However, a significantly lower rate of angiographic evaluation was observed in the late-pandemic group, suggesting that diagnostic or logistical constraints during the later stages of the pandemic may have affected clinical management. This finding is consistent with previous reports indicating that the COVID-19 pandemic disrupted routine neuroimaging and neurosurgical workflows in many healthcare settings worldwide [24][25].

Furthermore, while the pandemic (early vs. late) was not an independent predictor of mortality in multivariate analysis, the observed trends raise important concerns about the indirect effects of pandemic-related healthcare system strain, delayed referrals, and limited access to timely interventions for critical neurological conditions such as SAH.

Despite being well-established risk factors in aneurysm formation and rupture, variables such as hypertension history, diabetes, smoking, and alcohol consumption were not significantly associated with mortality in our study. This discrepancy might be attributed to underreporting in medical records or a limited sample size. Nonetheless, these factors should not be overlooked in risk assessments, especially in long-term outcome studies [26][27].

Our study confirms that early neurological assessment (GCS) and hemodynamic stabilization (BP control) are critical components in the management and risk stratification of SAH patients. These findings support current recommendations emphasizing rapid neurological evaluation and blood pressure monitoring during the acute phase [28][29].

This study has several limitations. Its retrospective design may introduce selection and information bias. Moreover, the lack of long-term follow-up data restricts our ability to evaluate delayed complications and functional outcomes. Additionally, the classification of pandemic impact based solely on admission date may not fully capture individual COVID-19 infection status or institutional variation in resources. Future multicenter prospective studies with larger cohorts and extended follow-up are necessary to validate these findings and develop predictive models. In conclusion, early neurological assessment and hemodynamic control remain pivotal in improving SAH outcomes, and healthcare preparedness during global crises is essential for maintaining critical care services.

## Conclusion

This study demonstrated that elevated systolic blood pressure, increased blood sugar levels at admission, reduced level of consciousness, higher Hunt and Hess scores, and lack of angiographic evaluation were significantly associated with in-hospital mortality among patients with non-traumatic subarachnoid hemorrhage. In contrast, variables such as age, gender, history of hypertension or diabetes, serum creatinine, and platelet count did not show significant associations with mortality. Although COVID-19 pandemic period was not independently associated with mortality in multivariate analysis, it was linked to reduced use of angiographic evaluation, which may have influenced patient outcomes indirectly. These findings underscore the importance of maintaining access to timely diagnostic and surgical interventions even during public health crises.

Further multicenter prospective studies are needed to clarify the long-term effects of the COVID-19 era on SAH outcomes and to optimize strategies for stroke care delivery in similar future emergencies.

## Conflict of Interest

The authors declare no conflict of interest.
